# Evaluation of the Effect of Nanographene Oxide on Microleakage of Conventional and Resin-Modified Glass Ionomer

**DOI:** 10.1155/2023/8853495

**Published:** 2023-11-09

**Authors:** Farahnaz Sharafeddin, Parisa Ghodrati

**Affiliations:** ^1^Department of Operative Dentistry, Biomaterials Research Center, School of Dentistry, Shiraz University of Medical Sciences, Shiraz, Iran; ^2^Department of Operative Dentistry, School of Dentistry, Shiraz University of Medical Sciences, Shiraz, Iran

## Abstract

**Objectives:**

One of the important features of the restorative materials is the ability to seal and prevent the microleakage. Glass ionomer cement (GIC) still exhibits some microleakage despite establishing a chemical bond to the tooth. The aim of this study was to evaluate the effect of nanographene oxide (nGO) on the microleakage of conventional (CGIC) and resin-modified glass ionomer cement (RMGIC).

**Methods:**

Thirty intact extracted molars were used. Class V cavities were prepared on their buccal and lingual surfaces. The samples randomly divided into two main groups of CGIC and RMGIC; each of them was randomly subdivided into three subgroups, including the group without nGO (control), the group with 1% nGO, and the group with 2% nGO. After restoring the cavities, they were subjected to thermocycling (1,000 cycles at 5/55°C). Two percent basic fuchsin solution was used to perform the microleakage test, and then the sectioned samples were examined by a stereomicroscope 40x. Kruskal–Wallis test, Dunn's test, and Mann–Whitney *U* test were used to analyze the data (*P* < 0.05).

**Results:**

Group CGIC + 1% nGO at the gingival margin and group RMGIC + 1% nGO at both gingival and occlusal margins had significantly less microleakage than their control groups (*P*=0.008, *P*=0.002, *P*=0.023, respectively). Also, in these two groups, there were no significant differences between the microleakage of the occlusal and gingival margins (*P*=0.132, *P*=0.511, respectively), while in all other groups, the gingival microleakage was significantly higher than that of occlusal microleakage.

**Conclusions:**

The addition of 1% nGO significantly reduced the gingival microleakage of CGIC and the occlusal and the gingival microleakage of RMGIC, while the addition of 2% nGO did not cause a significant reduction in microleakage.

## 1. Introduction

The goal of restorative dentistry is to restore the tooth in such a way that its shape and function are restored [1]. Class V lesions occur in the cervical area of the buccal or lingual surface of the anterior and posterior teeth. In general, the etiology of these lesions is divided into two categories: caries and noncaries [2, 3]. Until now, restorative materials such as conventional glass ionomer cement (CGIC), resin-modified glass ionomer cement (RMGIC), composite resins, and compomers have been used to repair class V lesions [4]. A key factor for the long-term success of the restorative materials is adhesion to the prepared cavity walls in order to seal the cavity against the microleakage [5]. Microleakage means the entry of bacteria, oral fluids, molecules, ions, and even air through the microgaps between the restorative material with incomplete adhesion and the walls of the cavity, which eventually causes secondary caries and increases sensitivity after treatment and pulp infection [6].

CGIC has been widely used due to its good chemical bond to the tooth, easy application, low cost, low thermal expansion coefficient, inherent adhesion to the dentin and enamel, and long-term release of fluoride; however, such reasons as fragility, poor resistance to crack propagation, and poor wear resistance have limited the clinical application of this cement [7]. While having properties such as adhesion to the enamel and dentin tissue, RMGIC offers better mechanical strength and a smoother surface than CGIC [8].

The need to improve the mechanical properties of the glass ionomer cement (GIC) has increased research on this material [9]. To improve the mechanical and physical properties of GICs, various materials such as metal powders, glass fibers, silica hydroxyapatite, and zirconia fillers have been used [10]. In recent years, attempts have been made to combine graphene-derived nanomaterials with commercially available GICs to strengthen them [11, 12]. Graphene is a two-dimensional material arranged in a honeycomb lattice, which consists of crystalline sp^2^-carbon atoms [13]. Graphene-based materials are thermally and chemically stable, retain a high surface area, and have premier mechanical properties. Graphene oxide (GO) can be obtained through graphite oxidation. Although graphene is a hydrophobic material, GO is considered hydrophilic as it contains oxygen in its functional groups [14]. GO is used in a variety of research experiments, for example, for teeth whitening, antimicrobial activity, dental erogenous, dental implants, tooth pain, and drug delivery in a specific location [15].

Recent research has shown that adding nanoparticles such as nanographene oxide (nGO) to GICs improves the physical, mechanical, and antibacterial properties of this cement due to their high specific surface area and two-dimensional structure [16–19]. Mei et al. [20] showed in a similar study that the addition of 1% graphene oxide-silica particles led to the improvement of the compressive strength of experimental adhesives. So far, research has been done on the effect of adding different percentages of GO, including 0.25%, 0.5%, 1%, 2%, and 4%, on the bond strength of dental adhesives and GICs to the dentin [17]. As shown in previous studies, GO improves the bond strength of adhesives and GICs to the tooth [20]; it can be assumed that it has the potential to affect their microleakage as well.

Since no extensive study has been conducted on the effect of adding GO on the microleakage of GICs, in the present study, the effect of adding nGO on the microleakage of CGIC and RMGIC was investigated. It was hypothesized that the addition of 1 and 2 wt% of nGO to CGIC and RMGIC does not reduce their microleakage.

## 2. Materials and Methods

### 2.1. Preparation of the Teeth

In this experimental study, after approval of the study with the ethics code of IR.SUMS.DENTAL.REC.1401.030, 30 intact human mandibular third molar teeth with no decay, repair, or fracture were used. All the teeth had been extracted because of orthodontics reasons. After extraction, the teeth were washed with water and the debris was removed by ultrasonic scaler. Then, they were stored in the solution of 1% thymol in distilled water at 4°C up to 1 month after extraction. Class V cavities were prepared on the buccal and lingual surfaces of the teeth (5 mm length, 3 mm width, and 2 mm depth) by a diamond fissure bur (Diamond Fissure 330, SS White, Washington, USA) in a high-speed handpiece and under water cooling. The bur was replaced with a new one after cutting all five cavities. The occlusal edge of class V cavities was placed at the enamel and their gingival edge was 1 mm below the cementoenamel junction (CEJ) at the dentin. The dimensions of the cavities were confirmed using a periodontal probe. The cavities were conditioned with 10% polyacrylic acid (GC Corporation, Tokyo, Japan) for 20 s and then gently washed with water and dried with a cotton pellet but not desiccated.

### 2.2. Research Groups

The samples were randomly divided into two main groups of CGIC and RMGIC (GC Corporation, Tokyo, Japan); the main group of CGIC was randomly subdivided into three subgroups (*n* = 10): group 1 (CGIC), group 2 (CGIC + 1 wt% nGO), and group 3 (CGIC + 2 wt% nGO). The main group of RMGIC was divided into three subgroups in the same way (*n* = 10): group 1 (RMGIC), group 2 (RMGIC + 1 wt% nGO), and group 3 (RMGIC + 2 wt% nGO).

### 2.3. Addition of nGO to the Powder of GICs

One weight percent and 2 wt% of nGO (US Research Nanomaterials, Inc., Houston, USA) were added to the powder of CGIC and RMGIC using a digital scale (A&D, GR + 360, Tokyo, Japan) with an accuracy of 0.0001 g. The powder was mixed using a mixing spatula. After that, to make the uniform mixture, the prepared powder was poured into the empty and clean amalgam capsule and vibrated for 20 s in the amalgamator (Faghihi, FD-4300, Iran). Then, in order to ensure uniform mixing, we examined the powder under a stereomicroscope (BestScope, BS-3060C, China) with 40x magnification [21].

### 2.4. Filling the Cavities

To mix the powder with the liquid according to the manufacturer's instructions, one scoop of powder was mixed with a drop of liquid for groups of CGIC, and one scoop of powder was mixed with two drops of liquid for RMGIC groups. Mixing of powder with liquid was done on a clean slab by a plastic spatula [22], and it was inserted inside the prepared class V cavities using a thin composite instrument. In the CGIC groups, it took 5 min and 30 s to set completely, and groups of RMGIC were cured for 20 s by the LED light curing device (BlueLEX, Monitex, Taiwan) with a light intensity of 1,200 mW/cm^2^ according to the manufacturer's instruction at a distance of 1 mm from the surface of the cement. A transparent matrix was adapted over the cement during its setting; after the setting was completed, a layer of self-cured varnish (GC Corporation, Tokyo, Japan) was applied on the surface of the cement. All the samples were stored in an incubator (Nuve, Turkey) with a temperature of 37°C and humidity of about 100% for 24 hr. Then, the surface of all the restorations was finished and polished carefully using the standard finishing and polishing disks (Sof-Lex Discs, 3M Dental Products). The teeth were mounted in the self-hardening acrylic resin (Acropars, Iran) in a cylindrical mold (with a diameter of 30 mm and a height of 30 mm up to the 3 mm below the CEJ). To simulate the oral environment condition, thermocycling was carried out (PC300; Vafaei, Iran) for 1,000 cycles at 5/55°C with a dwell time of 30 s and with a transfer time of 30 s.

### 2.5. Microleakage Test

All the surfaces of the teeth except the 1 mm border around the restoration were covered with two layers of nail polish. The teeth were immersed in the 2% basic fuchsin dye solution (Merck, Germany) for 24 hr. Then, the teeth were removed from the fuchsin solution, and the superficial discoloration was washed by the water flow. In order to evaluate the dye penetration, we sectioned the teeth horizontally at the top of the acrylic surface and longitudinally in the buccolingual direction at the midpoint of the restoration by a diamond disk (Diamond Disk, Microdont, Brazil) in a nonstop cutting machine (Demco E96, CMP Industries, NY, USA) under the water cooling spray. The sectioned surfaces were examined in random order using a stereomicroscope (BestScope, BS-3060C, China) with 40x magnification by two examiners who were unaware of the type of the restorative cement. Dye penetration along the tooth–restoration interface was recorded at the occlusal and the gingival margins according to the following scores:

0: No dye penetration.

1: Dye penetration less than half of the distance between the tooth surface and the axial wall.

2: Dye penetration more than half of the distance between the tooth surface and the axial wall but no axial wall involvement.

3: Dye penetration involving the axial wall of the cavity [23].

If there was disagreement between the examiners, consensus was obtained after re-examination of the specimen by both examiners.

### 2.6. Statistical Analysis

The data obtained from this research were statistically analyzed using SPSS software (IBM statistics version 26). Kruskal–Wallis test, Dunn's test, and Mann–Whitney *U* test were used to compare the microleakage between groups and between occlusal and gingival margins. A *P*-value < 0.05 was considered as statistically significant.

The information about the materials that have been used in this study are shown in Table 1.

## 3. Results

**Figure 1:**
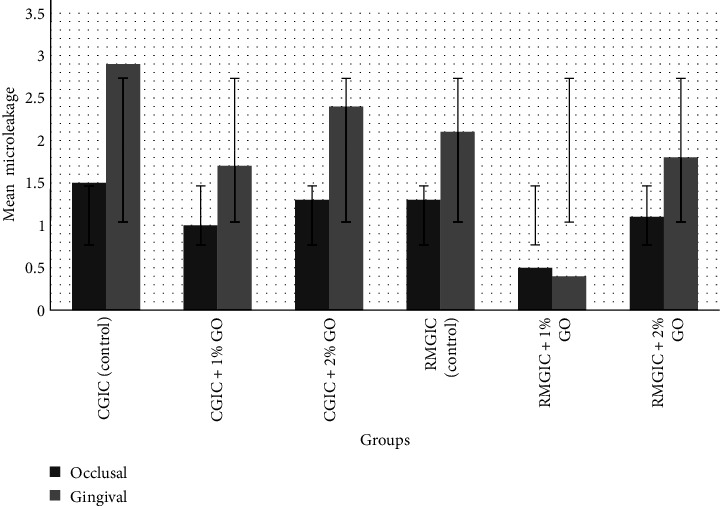
Mean and standard deviations of occlusal and gingival microleakage.

The means and standard deviations (SD) of the microleakage scores are shown in Tables 2–4 and Figure 1. According to the Kruskal–Wallis test, the difference in the microleakage of the occlusal margin of CGIC groups, unlike gingival, was not significant (*P*=0.326, *P*=0.011, respectively). Pairwise comparison by Dunn's test showed that at the gingival margin, the microleakage of CGIC + 1% nGO was significantly lower than the control CGIC (*P*=0.008), but CGIC + 2% nGO had no significant difference with the control CGIC and CGIC + 1% nGO (*P*=0.201, *P*=0.743, respectively). Moreover, at the occlusal margin, the microleakage of RMGIC + 1% nGO was significantly lower than the control RMGIC (*P*=0.023), while RMGIC + 2% nGO was insignificantly different from the control RMGIC and RMGIC + 1% nGO (*P*=1.000, *P*=0.131, respectively). At the gingival margin, RMGIC + 1% nGO was significantly lower than the control (RMGIC) and RMGIC + 2% nGO (*P*=0.002, *P*=0.014, respectively), but the difference of RMGIC + 2% nGO with the control (RMGIC) was insignificant (*P*=1.000).

The Mann–Whitney *U* test showed that in all groups, gingival microleakage was significantly higher than that of occlusal, except for groups CGIC + 1% nGO and RMGIC + 1% nGO, where the difference was not significant (*P*=0.132, *P*=0.511, respectively). Only in RMGIC + 1% nGO, the gingival microleakage was insignificantly lower than the occlusal microleakage (*P*=0.511).

Microleakage of the occlusal margin of RMGIC groups was not significantly different from their corresponding groups in CGIC, but at the gingival margin, the control RMGIC and RMGIC + 1% nGO had significantly lower microleakage compared to their corresponding CGIC groups (*P*=0.007, *P*=0.012, respectively).

**Figure 2:**
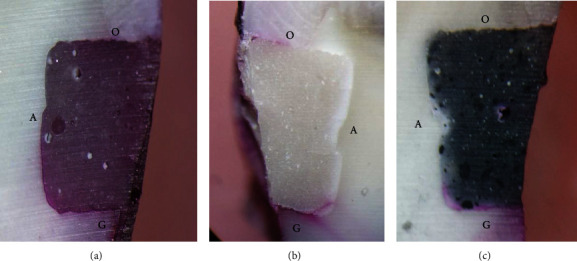
The images of the samples under the stereomicroscope with 40x magnification. (a) Dye penetration with the score of 2 at the occlusal margin and the score of 3 at the gingival margin. (b) Dye penetration with the score of 1 at the occlusal margin and the score of 3 at the gingival margin. (c) Dye penetration with the score of 0 at the occlusal margin and the score of 3 at the gingival margin (O, occlusal margin; G, gingival margin; A, axial wall; the scores of dye penetration: 0 = no dye penetration; 1 = dye penetration less than half of the distance between the tooth surface and the axial wall; 2 = dye penetration more than half of the distance between the tooth surface and the axial wall, but no axial wall involvement; 3 = dye penetration involving the axial wall of the cavity).

The images of the some samples under the stereomicroscope with 40x magnification and their scores are shown in Figure 2.

## 4. Discussion

In various studies, the effect of adding different weight percentages of GO, including 0.25%, 0.5%, 1%, 2%, 4%, and 5%, to adhesives and GIC has been investigated. Since the improvement of mechanical and physical properties such as compressive strength, shear bond strength, and flexural strength of GICs has been observed by adding 0.5, 1, and 2 wt% nGO [17, 18, 24, 25], therefore, in the present study, the effect of adding 1% and 2% by weight of nGO on the microleakage of CGIC and RMGIC was investigated. Two percent fuchsin dye penetration technique was used to evaluate microleakage due to its convenient and cost-effective application [22].

According to the results, comparing the microleakage of the occlusal margin of the CGIC groups showed that the addition of 1% and 2% nGO caused a nonsignificant decrease in the microleakage compared to the control CGIC group, while the gingival margin of the group containing 1% nGO had significantly less microleakage compared to the control CGIC group. However, in RMGIC groups, at both occlusal and gingival margins, addition of 1% nGO caused a significant reduction of the microleakage compared to the control RMGIC group. Therefore, the research hypothesis was relatively rejected. According to previous studies, GO has various functional groups, which help to improve its bond to the organic polymers, thus improving the mechanical properties of restorative materials [18]. Also, GO nanoparticles can increase their bond strength to the dentin due to their specific surface area and two-dimensional structure, if they are uniformly distributed in restorative materials [17]. It seems that improving the bond strength of the restorative material to the tooth tissue leads to a decrease in the microleakage [22]. In addition, GO has a hydrophilic nature due to the presence of functional groups such as hydroxyl and carboxyl, which improves the wetting ability and, as a result, better penetration of the cement into the intertubular spaces of the dentin [26]. In the present study, it can be assumed that the addition of 1% nGO to GICs reduces the microleakage by creating a higher quality seal at the interface between the tooth and the cement.

Meanwhile, the addition of 2% nGO compared to 1% in both CGIC and RMGIC groups and at both occlusal and gingival margins showed more microleakage, which could occur for several reasons. Since GO nanoparticles tend to stick to each other and form clusters, increasing the amount of these fillers by increasing the surface interaction makes the cement more viscous [27]. Also, the nonuniform distribution of the fillers in the cement leads to the creation of aggregated masses of nanoparticles and, as a result, the formation of porosities and microcracks in the cement [24]. These porosities can be created both between the cement and the tooth and in the cement mass and cause the weakness of the structure and integrity of the cement. These pores and cracks can also increase the microleakage [4]. According to the results of our study, it seems that although lower concentrations of GO due to the hydrophilic nature of this filler improve the wetting ability and penetration of the cement into the intertubular spaces of the dentin [26], higher concentrations of GO can cause more water absorption and destroy the bond between the cement and the tooth [28], thus increasing the microleakage.

In the restorative materials with free radical polymerization that must be light-cured, increasing the amount of fillers, especially if they are the same size as the wavelength of the curing light, acts as a barrier against the light penetration and scatters it, which in turn reduces the degree of conversion (DC) of this category of materials [29]. In a study, it was shown that increasing the amount of nGO from 0.5% to 2% by weight caused a significant decrease in its DC due to the adhesion of nGO plates to each other and the formation of aggregated masses that prevented the passage of the curing light [26]. In our research, addition of 2 wt% nGO compared to 1% in the RMGIC caused a significant increase in the microleakage at the gingival margin. It can be assumed that the higher amounts of nGO by reducing the DC of the resin part of RMGIC will cause its incomplete polymerization and, as a result, greater solubility of the cement [30], which could increase the microleakage.

According to our results, the occlusal microleakage of RMGIC groups was insignificantly lower than their corresponding groups in CGIC, which is in agreement with some previous studies [31]. However, the gingival microleakage of the control RMGIC group and RMGIC + 1% nGO was significantly lower than their corresponding groups in CGIC, as this result was seen in other studies [32]. RMGIC has a higher bond strength to the dentin compared to the CGIC. The reaction of the free radical polymerization and light curing improves the micromechanical bonding of the RMGIC to the dentin; in addition, the presence of the hydrophilic monomer hydroxyethyl methacrylate (HEMA) in RMGIC causes a better wetting ability and improves its mechanical and chemical adhesion to the dentin [23]. Moreover, in the present study, 10% polyacrylic acid was used to condition the surface of the cavity, which itself caused the partial demineralization of the dentin surface, which improved the penetration and diffusion of RMGIC to the intertubular spaces of the dentin and establishing a micromechanical bond to the dentin [33]; probably in this way, it has caused a reduction of the microleakage. However, this may not be the case with CGIC because the adhesion of CGIC which does not have a resin part to the dentin is chemical, and it can be estimated that the use of 10% polyacrylic acid has caused a decrease in the content of the minerals in the dentin, and, probably, it has a negative effect on the bond strength [34] and microleakage.

In any case, it is necessary to mention that obtaining different results in different studies [31, 32, 34] regarding the comparison of the microleakage of CGIC and RMGIC could be due to the study design, materials used, quality of the substrates, location of the margins, and different measurement tests.

Comparing microleakage of the occlusal margin with the gingival margin showed that in all groups of this study, except for the groups containing 1% nGO, the values of microleakage at the gingival margin were significantly higher than that of the occlusal ones, which were in line with some previous studies [4, 23, 35]. In general, the enamel compared to the dentin has a higher mineral content of hydroxyapatite (90%–92% and 50% by volume, respectively) [36]. CGIC is able to establish a chemical bond between the carboxyl groups of polyacrylic acid and hydroxyapatite on the surface of the tooth. Therefore, lower microleakage of the enamel margin can be attributed to the more effective adhesion of the cement to the enamel due to its greater hydroxyapatite content [23].

The temperature changes created in the dynamic environment of the mouth due to the difference in the coefficient of thermal expansion of the restorative material and tooth tissue lead to destruction of the bond at their interface [29]. In this study, the simulation of stress caused by temperature changes in the oral environment was done by using thermocycling (1,000 cycles at 5/55°C). In general, the more the number of the cycles, the more the bond destruction. Because the bond to the dentin is weaker than that to the enamel due to the more organic content, it is more damaged during the thermocycling process and causes increased microleakage at the gingival margin compared to the occlusal margin. However, in groups CGIC + 1% nGO and RMGIC + 1% nGO, gingival and occlusal microleakage did not have significant differences. Therefore, in this study, addition of 1% nGO reduced the microleakage of CGIC and RMGIC, especially in the gingival margin.

Among the limitations of this study, we can mention the lack of reconstruction of different clinical conditions in the oral environment, such as the effect of food acidity and chewing and brushing forces. It is suggested that more studies should be done with emphasis on different concentrations of GO and different microleakage measurement tests to achieve more reliable results.

## 5. Conclusion

Despite the limitations of this study, it was concluded that:

1. The addition of 1 wt% nGO significantly reduced the gingival microleakage of CGIC and the occlusal and gingival microleakage of RMGIC.
2. Addition of 2 wt% nGO did not significantly reduce neither occlusal nor gingival microleakage of CGIC and RMGIC.
3. The gingival microleakage was significantly more than occlusal, except in the RMGIC + 1 wt% nGO.

## Figures and Tables

**Table 1:** Information about the materials used.

Materials	Composition	Manufacturers
Conventional glass ionomer cement, Fuji II	Powder: fluoroaluminosilicate glass; liquid: polyacrylic acid, itaconic acid, tartaric acid, maleic acid, water	GC Corporation, Tokyo, Japan

Resin-modified glass ionomer cement, Fuji II LC	Powder: fluoroaluminosilicate glass; liquid: polyacrylic acid, 2-hydroxylethyl methacrylate, urethane dimethacrylate, camphorquinone, distilled water	GC Corporation, Tokyo, Japan

Dentin conditioner	10% polyacrylic acid	GC Corporation, Tokyo, Japan
Nanographene oxide powder	Graphene oxide nanoplatelets	US Research Nanomaterials, Inc., Houston, USA

GC Fuji Varnish	Isopropyl acetate 50%–70% and acetone 20%–30%	GC Corporation, Tokyo, Japan

**Table 2:** Mean ± SD of microleakage in CGIC subgroups.

Groups	Occlusal mean ± SD	Gingival mean ± SD	^*∗*^*P*-value
CGIC (control)	1.5 ± 0.70^a^	2.9 ± 0.31^a^	0.001
CGIC + 1% nGO	1.0 ± 0.66^a^	1.7 ± 1.15^b^	0.132
CGIC + 2% nGO	1.3 ± 0.67^a^	2.4 ± 0.51^ab^	0.002
^*∗*^ ^*∗*^*P*-value	0.326	0.011	

^*∗*^Mann–Whitney *U* test,  ^*∗*^ ^*∗*^Kruskal–Wallis test. In each column, mean values with at least the same letter were not statistically significant (Dunn's test). CGIC, conventional glass ionomer cement; nGO, nanographene oxide.

**Table 3:** Mean ± SD of microleakage in RMGIC subgroups.

Groups	Occlusal mean ± SD	Gingival mean ± SD	^*∗*^*P*-value
RMGIC (control)	1.3 ± 0.67^a^	2.1 ± 0.73^a^	0.028
RMGIC + 1% nGO	0.5 ± 0.52^b^	0.4 ± 0.69^b^	0.511
RMGIC + 2% nGO	1.1 ± 0.56^ab^	1.8 ± 1.03^ac^	0.045
^*∗*^ ^*∗*^*P*-value	0.021	0.001	

^*∗*^Mann–Whitney *U* test,  ^*∗*^ ^*∗*^Kruskal–Wallis test. In each column, mean values with at least the same letter were not statistically significant (Dunn's test). RMGIC, resin-modified glass ionomer cement; nGO, nanographene oxide.

**Table 4:** Occlusal and gingival microleakage of CGIC groups compared to their corresponding groups in RMGIC.

Groups	^*∗*^*P*-value	
	Occlusal	Gingival
CGIC (control) - RMGIC (control)	0.671	0.007
CGIC + 1% nGO - RMGIC + 1% nGO	0.089	0.012
CGIC + 2% nGO - RMGIC + 2% nGO	0.435	0.165

^*∗*^Mann–Whitney *U* test. CGIC, conventional glass ionomer cement; RMGIC, resin-modified glass ionomer cement; nGO, nanographene oxide.

## Data Availability

The data supporting this research article were represented at the time of submission.
